# Interpreting Performance of Deep Neural Networks with Partial Information Decomposition

**DOI:** 10.3390/e28010050

**Published:** 2025-12-31

**Authors:** Tianyue Liu, Binghui Guo, Ziqiao Yin, Zhilong Mi, Donghui Jin

**Affiliations:** 1 School of Artificial Intelligence, Beihang University, Beijing 100191, China; liutianyue@buaa.edu.cn (T.L.);; 2 Zhongguancun Laboratory, Beijing 100194, China; 3 LMIB and SKLCCSE, Beihang University, Beijing 100191, China; 4 Beijing Advanced Innovation Center for Future Blockchain and Privacy Computing, Beijing 100083, China; 5 Hangzhou Internation Innovation Institute of Beihang University, Hangzhou 311115, China

**Keywords:** partial information decomposition, model interpretation, corruption robustness

## Abstract

Robustness to distributional shifts remains a critical limitation for deploying deep neural networks (DNNs) in real-world applications. While DNNs excel in standard benchmarks, their performance often deteriorates under unseen or perturbed conditions. Understanding how internal information representations relate to such robustness remains underexplored. In this work, we propose an interpretable framework for robustness assessment based on partial information decomposition (PID), which quantifies how neurons redundantly, uniquely, or synergistically encode task-relevant information. Analysis of PID measures computed from clean inputs reveals that models characterized by higher redundancy rates and lower synergy rates tend to maintain more stable performance under various natural corruptions. Additionally, a higher rate of unique information is positively associated with improved classification accuracy on the data from which the measure is computed. These findings provide new insights for understanding and comparing model behavior through internal information analysis, and highlight the feasibility of lightweight robustness assessment without requiring extensive access to corrupted data.

## 1. Introduction

In recent years, deep neural networks (DNNs) have achieved remarkable success in various domains such as image recognition [1], recommendation systems [2], and natural language processing [3]. However, their performance heavily relies on the distribution of training data [4], making them vulnerable to distributional shifts between training and test domains [5]. This issue is particularly critical in autonomous driving, where complex and dynamic environments introduce natural corruptions such as illumination changes, adverse weather, and blur or defocus of images, all of which can severely impair visual perception models [6]. Such degradations not only reduce model accuracy but may also lead to serious safety failures, making corruption robustness a key prerequisite for deploying DNNs in autonomous driving systems. Empirical evidence shows that mainstream DNNs often experience substantial accuracy degradation when exposed to such corruptions [7]. Although augmenting training data with corrupted samples may improve robustness, it often leads to overfitting to seen corruption types, limiting generalization to unseen degradation patterns [8]. These challenges underscore the need for reliable corruption robustness evaluation and a deeper understanding of its structural foundations. Mainstream corruption robustness evaluation methods typically assess model performance degradation under naturally corrupted inputs using benchmark datasets [9], or quantify sensitivity through output fluctuations and confidence shifts [10]. However, these approaches often require large volumes of corrupted data and are computationally expensive. More importantly, being based on posterior observations, they offer limited insight into the intrinsic relationship between a model’s internal information structure and its robustness.

Information theoretic approaches have increasingly informed the theoretical understanding of DNNs [11]. Researchers have sought to characterize how information is transmitted and transformed across neural layers using concepts such as mutual information and entropy, aiming to uncover their intrinsic links to generalization, representational efficiency, and robustness. The Information Bottleneck (IB) theory [12] provided an early perspective by framing deep learning as a trade-off between input compression and task-relevant retention, with subsequent studies linking compression dynamics to generalization [13,14].

Building on this, partial information decomposition [15] (PID) has emerged as a refined multivariate framework that decomposes mutual information into redundant, synergistic, and unique components, enabling deeper insights into how neurons share, integrate, and specialize information. PID has been applied to both biological and artificial neural systems. In neuroscience, Luppi et al. [16] used PID to map the brain’s informational architecture, while Varley et al. [17] employed partial entropy decomposition to uncover higher-order structures in human activity. In artificial systems, Wollstadt et al. [18] defined redundancy and relevancy for feature selection, Wibral et al. [19] proposed PID as a unified framework for neural goal functions, and Dewan et al. [20] applied Diffusion PID to interpret generative models. Together, these studies highlight PID’s role in elucidating information dynamics, refining feature selection, and advancing model interpretability. Recent studies have begun to investigate the functional roles of the components defined by PID. Proca et al. [21] applied PID to simple neural networks across supervised and reinforcement learning tasks, finding that synergistic information supports multimodal integration and multitask learning, while redundancy correlates with robustness, highlighting the role of internal information dynamics in general learning ability. Moreover, Tax et al. [22] used PID to analyze hidden neuron representations in Boltzmann machines, revealing a staged learning pattern from redundancy to uniqueness. While these findings provide empirical support for the functional significance of PID components, they are mostly derived from small-scale networks and have not yet been extended to more complex architectures or realistic tasks.

Inspired by the aforementioned studies, this work investigates how the structure of neuronal information interactions relates to model performance, with particular emphasis on robustness under natural corruptions. We introduce a robustness assessment approach based on PID measures, aiming to estimate a model’s stability across corruption scenarios solely from the information structure revealed by its neural activations on clean images. Just as network depth is widely seen as a structural indicator of model expressiveness [23], we explore whether information structure can likewise serve as a prior for robustness, enabling efficient model assessment and comparison. Our main contributions are: (1) we reveal how redundant, synergistic, and unique information components differentially account for performance variation under corruption; (2) through empirical analysis on two benchmark corruption datasets and six mainstream architectures, we validate the potential of inferring robustness using PID metrics computed from clean samples, providing theoretical insights into model assessment and design.

## 2. Partial Information Decomposition

In information theory, Mutual Information (MI) [24] is a fundamental measure used to quantify the dependence between two random variables. For two discrete random variables *X* and *Y*, mutual information is defined as follows:

(1) $$I\left(X;Y\right)=\sum_{x\in X}\sum_{y\in Y}\;p\left(x,y\right)\log\frac{p\left(x,y\right)}{p\left(x\right)p\left(y\right)},$$

where p(x,y) denotes the joint probability distribution of *X* and *Y* and p(x), p(y) are the marginal distributions. MI can also be equivalently expressed in terms of entropy:

(2) $$I\left(X;Y\right)=H\left(Y\right)-H\left(Y|X\right),$$

here, H(Y) represents the Shannon entropy of *Y*, quantifying the information content of the variable:

(3) $$H\left(Y\right)=-\sum_{y\in Y}\;p\left(y\right)\log p\left(y\right).$$

The term H(Y|X), known as conditional entropy, measures the remaining uncertainty in *Y* given knowledge of *X*:

(4) $$H\left(Y|\;X\right)=-\sum_{x\in X}\sum_{y\in Y}\;p\left(x,y\right)\log p\left(y|\;x\right).$$

MI thus measures the reduction in uncertainty about *Y* given knowledge of *X*, i.e., how much information *X* provides about *Y*. However, in multivariate settings, MI cannot distinguish between different types of contribution from multiple sources, for example, whether they offer redundant, unique, or synergistic information about the target variable. To address this limitation, PID provides a framework for decomposing mutual information into interpretable atomic components.

PID, introduced by Williams and Beer [15], extends Shannon’s framework to analyze how multiple source variables jointly contribute to a target variable. Given a set of sources $X=\{X_{1},X_{2},\ldots ,X_{n}\}$ and a target *Y*, PID decomposes the total mutual information $I\left(X;Y\right)=H\left(Y\right)-H\left(Y|X\right)$ into the following fundamental components:

**Redundant Information**: Information that is shared by multiple source variables—i.e., the same information about *Y* is provided by more than one source.

**Unique Information**: Information that is exclusively provided by a single source variable and not available from any other sources.

**Synergistic Information**: Information that can only be obtained through the joint consideration of multiple source variables, which cannot be accessed from any individual source alone.

**Figure 1:**
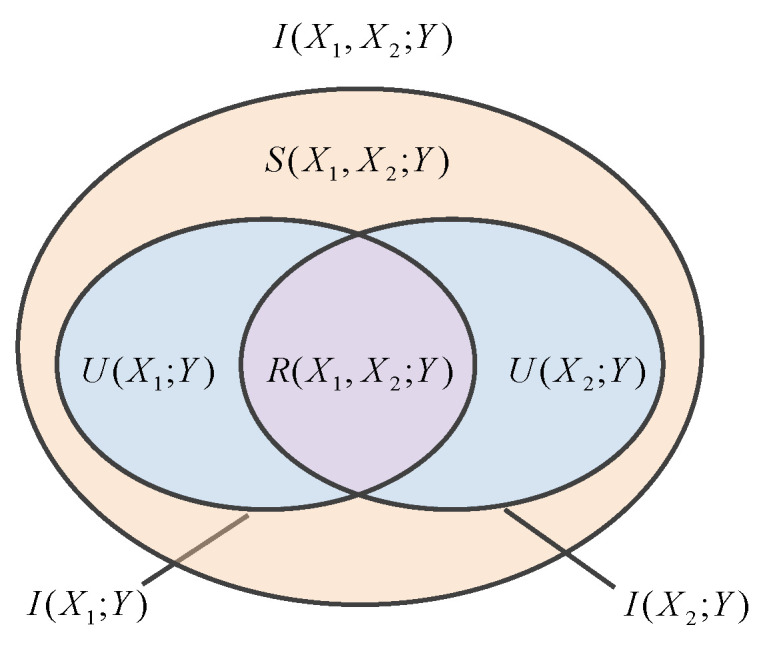
Partial information diagram.

To illustrate the decomposition more intuitively, consider a simplified case with two source variables $X_{1}$ and $X_{2}$, and a single target variable *Y*. The mutual information between the joint sources and the target can be decomposed into a sum of partial information atoms as shown in Equation (5), $I(X_{i};Y)$ denotes the mutual information between a single source $X_{i}$ and the target *Y*, and $I(X_{1},X_{2};Y)$ denotes the mutual information between the joint sources $\left(X_{1},X_{2}\right)$ and the target *Y*. $R(X_{1},X_{2};Y)$ represents the redundant information about *Y* shared by $X_{1}$ and $X_{2}$, $U(X_{i};Y)$ represents the information uniquely provided by $X_{i}$, and $S(X_{1},X_{2};Y)$ represents the synergistic information about *Y* that is only provided jointly by $X_{1}$ and $X_{2}$. The relationships among these components can be intuitively illustrated using a Venn diagram, as depicted in Figure 1.

(5) $$\left\{\begin{array}{cc} I(X_{1},X_{2};Y) & =R\left(X_{1},X_{2};Y\right)+U\left(X_{1};Y\right)+U\left(X_{2};Y\right)+S\left(X_{1},X_{2};Y\right)\\ I(X_{1};Y) & =R\left(X_{1},X_{2};Y\right)+U\left(X_{1};Y\right)\\ I(X_{2};Y) & =R\left(X_{1},X_{2};Y\right)+U\left(X_{2};Y\right) \end{array}\right.$$

This results in an underdetermined system of three equations with four unknowns. Although PID provides a conceptual framework to differentiate redundant, unique, and synergistic information, it does not prescribe a specific method for computing these quantities. Currently, there is no universally accepted redundancy function, and alternative formulations characterize distinct facets of multivariate information. Two commonly used redundancy functions are $I_{\min}$, which was originally proposed by [15], and $I_{MMI}$ [25]. As demonstrated in the study by [21], these two measures exhibit consistent behavior in various experimental settings. Therefore, for computational tractability, we adopt the $I_{\min}$ measure to compute the redundancy function, that is,

(6) $$R\left(X_{1},X_{2};Y\right)=\min\left\{I\left(X_{1};Y\right),I\left(X_{2};Y\right)\right\}.$$

## 3. Methods

### 3.1. Datasets and Models

This study utilizes the CIFAR10 and ImageNet datasets for experimental purposes. CIFAR10 consists of 10 object classes with images sized at 32 × 32 × 3. ImageNet comprises a larger scale image collection, for simplicity, 100 classes are selected from the 1000 categories available in the ILSVRC-2012 subset. Since the purpose of this work is to compare models’ classification performance and PID measures, the number of ImageNet classes used does not affect the relative behaviors of the models, and the conclusions remain generalizable.

To evaluate model performance under noisy and degraded conditions, ImageNet-C and CIFAR10-C corruption benchmark datasets are used, as introduced in [26]. These datasets simulate common types of real-world degradation, including four major corruption categories: noise, blur, weather, and digital distortions. Two specific corruption types are selected from each category, including Shot Noise, Gaussian Noise, Motion Blur, Defocus Blur, Snow, Fog, Pixelate and Contrast. Detailed descriptions of these corruption types are available in [26]. Each type of corruption contains five severity levels, denoted as *s*. Since images at s=5 are heavily degraded and typically render models ineffective, only levels 1 to 4 are included in this work. Uncorrupted (clean) images from the original ImageNet and CIFAR10 datasets are treated as s=0.

Six convolutional neural networks with diverse architectures are selected for analysis of information interaction characteristics: MobileNet V2 [27], AlexNet [28], VGG 16 [29], Inception V3 [30], ResNet 50 [31], and DenseNet 121 [32]. All models are initialized using pretrained weights provided in PyTorch 2.9.1 and are not fine-tuned on any corrupted data. Each model is evaluated in all types and severity levels, its performance and PID characteristics are analyzed accordingly. All experiments are conducted on a multi-core CPU platform equipped with dual Intel Xeon Gold-class processors. Under this setup, the PID analysis for a single model and dataset takes close to two hours.

### 3.2. Corruption Robustness

To evaluate model stability under image corruptions, this study adopts the corruption robustness definition proposed by [26], which is formally expressed as follows:

(7) $$E_{c\in C}\left[\underset{(x,y)∼D}{\mathrm{Pr}}\left(f(c(x))=y\right)\right],$$

where C denotes the set of corruption functions, D represents the distribution of clean image data, and *f* is the image classification model. Unlike adversarial robustness, which focuses on performance in the worst case, this formulation emphasizes performance on average under natural image degradations. To comprehensively assess model robustness, three evaluation metrics are employed.

**Mean corruption accuracy (mCA):** Let $A_{0}^{f}$ represent the model classification accuracy in clean images. For each type of corruption *c* and severity level *s*, the accuracy of the model is indicated as $A_{s,c}^{f}$. The specific accuracy of corruption is defined as the average over severity levels: ${CA}_{c}^{f}=1/4\sum_{s=1}^{4}A_{s,c}^{f}$. Averaging across all selected corruption types yields the mean corruption accuracy:

(8) $$mCA=\frac{1}{\left|C\right|}\sum_{c\in C}\left(\frac{1}{4}\sum_{s=1}^{4}A_{s,c}^{f}\right).$$

**Accuracy standard deviation ($\sigma_{\mathrm{Acc}}$):** it quantifies performance variability across clean and corrupted data and is defined as follows:

(9) $$\sigma_{Acc}=\mathrm{std}\left(\left\{A_{0}^{f}\right\}\cup \left\{A_{s,c}^{f}|s=1,\ldots ,4;\;c\in C\right\}\right),$$

specifically, $\sigma_{Acc}$ is computed as the standard deviation over the classification accuracy on clean data ($A_{0}^{f}$) together with the accuracies obtained under all considered corruption types c∈C and severity levels s=1,…,4, thereby measuring the dispersion of model performance across different evaluation conditions.

**Relative accuracy degradation (ΔAcc):** it measures the performance drop under corruption relative to clean data:

(10) $$\Delta Acc=\frac{mCA-A_{0}^{f}}{A_{0}^{f}}.$$

### 3.3. The Computation of PID

To investigate the relationship between the interaction of neuronal information and model performance, PID is performed on neuron activations obtained from the test datasets across various models. In information theoretic terms, the sources in PID can refer to individual neurons, specific input dimensions, or combinations of features treated as independent random variables. In this study, the sources are defined as the set of neurons within a given network layer. Specifically, we select neurons from the last pooling layer of each model, as this layer captures high level semantic representations that are critical to final decision-making [33]. The target variable is defined as the label of the ground truth class that corresponds to each input. During testing, sampled neuron activations are used to estimate the probability distributions of network activity, which are subsequently employed to quantify redundant, unique, and synergistic information shared between neuron pairs with respect to the classification target. This analysis enables a comparative analysis of the information interaction patterns across different models.

The PID framework is applicable to any number of source variables *K*, where *K* can represent the full set of neurons in a layer (full-order computation, denoted as PID-*K*) or be restricted to K=2 for second-order analysis (PID-2). In PID-2, information decomposition is performed only for neuron pairs, and the average of these pairwise decompositions is taken as the final metric. As the number of neurons increases, full-order decomposition over all neurons becomes computationally intractable. In addition, evaluating PID-2 over all neuron pairs can be computationally expensive for large layers. To address this, a uniform sampling strategy can be applied to efficiently approximate the second-order measurements. Moreover, prior work [21] has shown that second-order and full-order PID measures exhibit consistent qualitative behavior across different tasks. Based on these theoretical foundations, this study employs second-order PID analysis with uniform sampling, balancing computational feasibility and analytical reliability.

To analyze the information interaction among neurons within an information theoretic framework, following the approach in [21], the continuous activation values of neurons are discretized using a binning strategy that divides the range into 10 intervals of equal width, thereby transforming the continuous distributions into discrete probability distributions. 10 bins are used to ensure sufficient samples in each source–target pair for reliable estimation. Using the resulting discrete representations, the mutual information between the activation of a single neuron $X_{i}$ and the class label *Y*, denoted as $I(X_{i};Y)$, is computed. Likewise, for each sampled neuron pair $\left(X_{i},X_{j}\right)$, the joint mutual information $I(X_{i},X_{j};Y)$ is calculated.

With these mutual information values, partial information decomposition is applied according to Equations (5) and (6) to decompose the joint information into redundant information $R(X_{i},X_{j};Y)$, synergistic information $S(X_{i},X_{j};Y)$, and unique information $U_{1}\left(X_{i};Y\right)$ and $U_{2}\left(X_{j};Y\right)$. This decomposition enables the quantification of both individual and joint neuronal contributions to the classification task, thereby establishing a basis for analyzing the information processing mechanisms of different models. To ensure comparability across models and corruption types, we normalize the decomposed information by the total mutual information, yielding the relative measures of redundancy rate (RR), synergy rate (SR), and uniqueness rate (UR).

## 4. Results

### 4.1. Preliminary Observations on the Connection Between Neuronal PID Structure and Model Robustness

Each model is evaluated on the clean test set and eight corrupted test sets, with both prediction outputs and neuronal activations recorded. Partial information decomposition is subsequently applied to neuron pairs using clean image samples, in order to investigate potential relationships between internal information processing mechanisms and model behavior under corruption.

**Figure 2:**
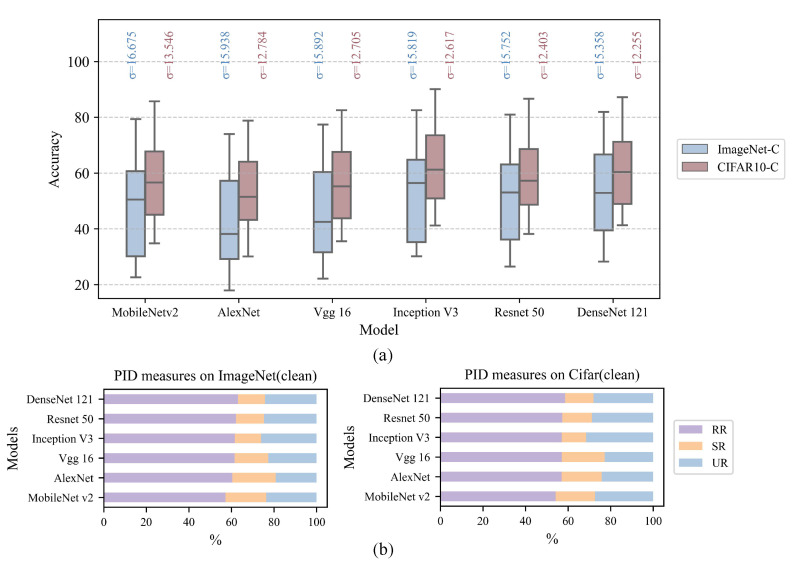
(**a**) Comparison of experimental results for different models; (**b**) PID measures on clean images (s=0). Models with smaller performance variability across corruption conditions tend to exhibit higher RR and lower SR values, whereas UR shows no consistent trend.

Figure 2a presents the classification accuracy of each model on the ImageNet-C and CIFAR10-C datasets across eight corruption types and five severity levels (0≤s≤4), together with the corresponding accuracy standard deviation $\sigma_{Acc}$, where a smaller $\sigma_{Acc}$ indicates less variation in accuracy across corruption types and severity levels. Figure 2b illustrates the PID results on clean images, including RR, SR, and UR. A comparison of the two subfigures indicates that models with smaller $\sigma_{Acc}$ tend to exhibit higher RR and lower SR values, whereas UR does not show a consistent trend. These observations provide preliminary evidence that the internal information structure of neurons may be related to model robustness under corruption.

### 4.2. Rank Correlation Analysis Between PID Measures and Robustness

To further assess the statistical validity of the trends observed in the previous section, a non-parametric rank correlation analysis is conducted to quantify the relationship between PID measures and model robustness. Unlike correlation metrics that rely on linear assumptions, Spearman’s ρ and Kendall’s τ are better suited for for capturing monotonic relationships, particularly when dealing with nonlinearity and ordinal consistency. Accordingly, this study applies both ρ and τ to systematically assess the rank correlation between three PID measures (RR, SR, UR) and the three robustness measures (mCA, $\sigma_{ACC}$, ΔAcc). This analysis aims to explore the potential of these metrics as predictors for model robustness.

**Figure 3:**
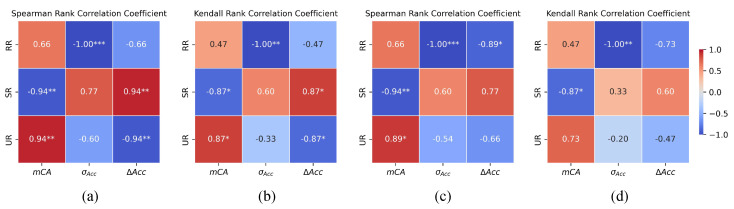
Rank correlations between PID measures on clean images (s=0) and robustness indicators on corrupted images. (**a**,**b**) show results on the ImageNet-C, (**c**,**d**) correspond to the CIFAR10-C. Significance levels are denoted by asterisks: * *p* < 0.05, ** *p* < 0.01, and *** *p* < 0.001.

The rank correlation results are presented in Figure 3. They reveal a consistent trend between the information decomposition metrics measured on images that s=0 and the robustness of models under corrupted conditions. Notably, RR exhibits a strong negative correlation with $\sigma_{ACC}$, with both Spearman’s ρ and Kendall’s τ reaching −1 and *p*-values below 0.01 across both datasets. This result indicates that models with higher redundancy tend to show smaller performance fluctuations across various corruption types and severities. This phenomenon can be attributed to the fault-tolerant properties of redundancy in neural networks. In scenarios where multiple neurons transmit overlapping information, the network remains stable even if part of the neurons is disrupted by noise or distortion, as the unaffected neurons compensate for the loss, thereby maintaining the overall stability of the system [34]. Similar conclusions were drawn by Barret et al. [25], who demonstrated that redundancy enhances a model’s resistance to external perturbations and ensures that critical information is robustly transmitted under varying input conditions.

SR exhibits a negative rank correlation with model robustness across both datasets. Specifically, models with higher SR tend to show greater fluctuations in accuracy under different types and severities of corruption, while the strength and statistical significance of this correlation vary between datasets, suggesting a less consistent association compared to RR. This observation can be explained by findings from [21], which suggest that neurons relying heavily on synergistic representations are more sensitive to input perturbations. Because synergistic information emerges through the combined activity of multiple sources, disruption to any one of these sources can compromise the integrity of the shared information. Consequently, the failure of such neurons to maintain stable information integration undermines the decision-making performance of model.

The differences in RR and synergy rate SR across models can be traced to their architectural design, reflecting how structural mechanisms shape internal information dynamics. ResNet50 and DenseNet121 consistently exhibit higher RR and lower SR, which can be attributed to their skip connections and dense feature reuse. These design choices promote overlapping information pathways, thereby enhancing redundancy and reducing reliance on fragile synergistic interactions. In contrast, AlexNet and MobileNetV2 show lower RR and higher SR, indicating that their relatively shallow or lightweight convolutional structures depend more on joint feature integration. Such reliance on synergy makes them more sensitive to corruption-induced perturbations, as disruption of any source neuron can compromise the shared representation. InceptionV3 demonstrates moderate RR but the lowest SR, suggesting that its multi-branch architecture fosters representational diversity while minimizing dependence on synergistic encoding. Finally, VGG16 lies in the middle range, with balanced RR and SR values, consistent with its deep yet sequential convolutional design that neither strongly emphasizes redundancy nor synergy. These observations highlight that redundancy and synergy rates reflect architectural design choices. Models with mechanisms that encourage overlapping information transmission tend to achieve greater robustness, whereas architectures that rely heavily on synergistic integration are more vulnerable to corruption.

UR shows a strong positive correlation with mCA, although the statistical significance is weaker in the CIFAR10-C dataset. Its rank correlation with ΔAcc is statistically significant on ImageNet-C but not on CIFAR10-C, and its correlations with $\sigma_{Acc}$ are not statistically significant on both datasets; therefore, the association between UR and robustness indicators is not consistent across datasets. This suggests that UR may be more involved in task-specific feature representation while playing a relatively limited role in general robustness mechanism.

In summary, these findings demonstrate that the PID measures of neurons, particularly redundancy and synergy, capture structural differences in how models respond to corrupted inputs. These results provide both theoretical support and empirical evidence for developing model evaluation approaches grounded in internal information structure.

### 4.3. Connection Between Unique Information and Classification Performance

**Figure 4:**
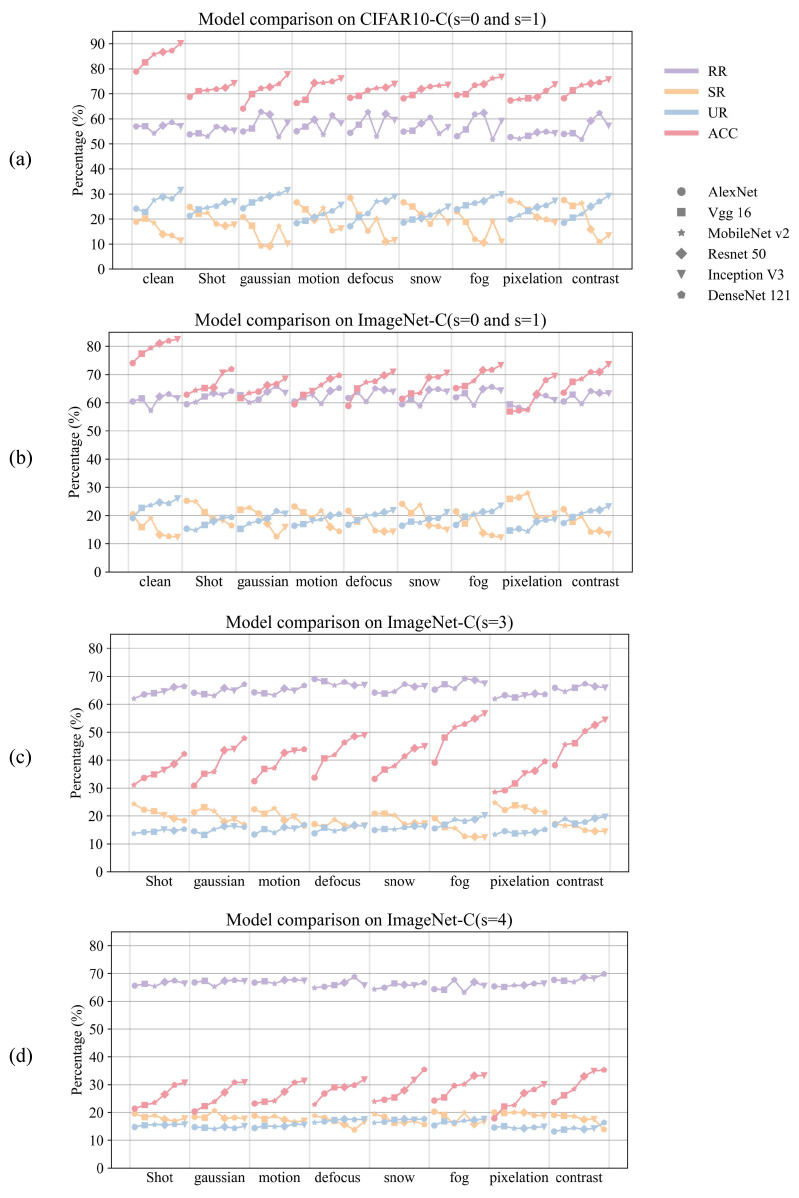
Classification accuracy and PID measures of six models on (**a**) CIFAR10-C (s=0 and s=1), (**b**) ImageNet-C (s=0 and s=1), (**c**) ImageNet-C (s=3), and (**d**) ImageNet-C (s=4). Models with higher accuracy generally exhibit higher UR under clean and mild corruption, whereas at severe corruption levels accuracy declines sharply and the correlation with UR becomes unclear.

Building upon the established association between neuronal information structure and model robustness, this section investigates how information interaction patterns relate to classification performance under varying corruption conditions. To this end, we evaluate model performance and compute PID measures on clean images (s=0) and mildly corrupted images (s=1) across eight corruption types in both ImageNet-C and CIFAR10-C. The results are illustrated in Figure 4a,b, where the x-axis represents corruption types and different markers denote distinct models. The results reveal that models achieving higher accuracy typically exhibit higher UR values, suggesting that unique information may reflect a model’s ability to extract discriminative features.

However, this trend weakens as the severity of corruption increases. Figure 4c,d presents the results for s=3 and s=4 on ImageNet-C, where the classification performance of all models declines significantly, and the differences in UR become increasingly disordered. At these higher corruption levels, accuracy approaches random behavior, as severe distortions overwhelm the useful signal, which obscures the correlation with UR and makes the trend less clear. This observation implies that UR may serve as a meaningful structural indicator of model capacity under high-quality input conditions, whereas under severe corruption, models may rely more on redundant mechanisms or other robustness strategies to maintain performance.

**Figure 5:**
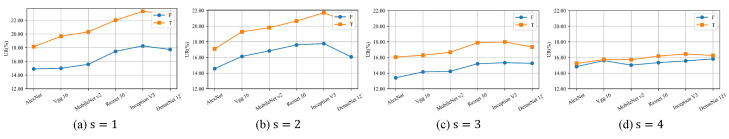
Comparison of UR between correctly and incorrectly classified samples on ImageNet-C. (**a**–**d**) correspond to corruption severity levels 1 through 4, respectively. Correctly classified samples consistently exhibit higher UR values than misclassified samples, with the difference most pronounced under mild corruption.

To further examine the discriminative capacity of unique information in classification tasks, UR were compared between correctly and incorrectly classified samples. As depicted in Figure 5, for corruption levels ranging from s=1 to s=4, correctly classified inputs consistently exhibit higher UR values than misclassified ones. This effect is particularly pronounced under mild corruption conditions, supporting the inference that increased unique information is linked to improved discriminative capability.

Theoretical support for this observation can be found in prior studies on the dynamics of information learning. Tax et al. [22] performed an analysis of neural information dynamics using the PID framework during the training of Boltzmann machines and observed that neurons first acquire redundant information before gradually specializing to encode unique information about the target variable. This transition reflects an increasing ability to capture discriminative features, thereby enhancing classification performance. From the perspective of statistical decision theory, Venkatesh et al. [35] further demonstrated that the amount of unique information provides an upper bound on the minimum risk achievable by a given information source in a decision task. In other words, neurons with higher UR contribute to lower uncertainty in classification decisions. Therefore, the positive relationship between UR and classification performance is supported by both empirical evidence and theoretical foundations.

The UR also reflects architectural design. InceptionV3, ResNet50, and DenseNet121 consistently show higher UR, indicating that multi-branch, residual, and dense connectivity promote feature specialization and discriminative capacity. MobileNetV2 exhibits intermediate UR, balancing efficiency with moderate uniqueness. In contrast, VGG16 and AlexNet display low UR, consistent with their sequential or shallow structures that rely more on shared representations. Overall, architectures that encourage diverse and specialized feature encoding achieve higher UR, while simpler sequential designs yield lower UR and weaker discriminative ability.

## 5. Conclusions

Within the framework of PID, the structure of neuronal information interactions is examined as a potential indicator of model robustness. PID measures derived from clean-image neuron activations are analyzed to explore the relationship between internal information interaction mechanisms and model robustness under corruption. This approach facilitates a novel robustness evaluation paradigm that does not rely on extensive corrupted test samples, thereby contributing both theoretical insights and practical value to model assessment.

Experiments conducted on the ImageNet-C and CIFAR10-C datasets reveal consistent trends in the connection between information decomposition and robustness. Models with a higher redundancy rate tend to achieve more stable performance across diverse corruption types and severity levels, whereas higher synergy rate is generally associated with increased performance variability. These results suggest that redundancy contributes critically to robustness by supporting tolerance to input degradation, while a greater dependence on synergistic information may heighten sensitivity to noise and perturbations. In addition, the unique information rate correlates with classification performance on high-quality inputs, indicating that unique information reflects the degree of specialization in encoding discriminative features. However, as corruption severity increases, the influence of unique information diminishes, and the network increasingly relies on redundancy to preserve performance.

Overall, our findings highlight the distinct functional roles of the components in PID. Redundancy is essential for robustness, synergy facilitates complex feature integration but introduces sensitivity, and uniqueness contributes to accuracy under high-quality input conditions. Future research should further investigate strategies for modulating the information structure of neural models to achieve a better balance between robustness and accuracy. For instance, specific architectural designs or training procedures could be developed to enhance redundancy and thereby improve stability under challenging conditions. Additionally, identifying methods to promote the learning of unique information without compromising robustness could be a promising direction toward building more interpretable neural networks.

## Data Availability

The data used in this study are publicly available [26].
